# The Noise Exposure Structured Interview (NESI): An Instrument for the Comprehensive Estimation of Lifetime Noise Exposure

**DOI:** 10.1177/2331216518803213

**Published:** 2018-10-08

**Authors:** Hannah Guest, Rebecca S. Dewey, Christopher J. Plack, Samuel Couth, Garreth Prendergast, Warren Bakay, Deborah A. Hall

**Affiliations:** 1 Manchester Centre for Audiology and Deafness, University of Manchester, Manchester Academic Health Science Centre, UK; 2 Sir Peter Mansfield Imaging Centre, School of Physics and Astronomy, University of Nottingham, UK; 3 NIHR Nottingham Biomedical Research Centre, Nottingham University Hospitals NHS Trust, UK; 4 Hearing Sciences, Division of Clinical Neuroscience, School of Medicine, University of Nottingham, UK; 5 NIHR Manchester Biomedical Research Centre, Central Manchester University Hospitals Foundation Trust, UK; 6 Department of Psychology, Lancaster University, UK; 7 University of Nottingham Malaysia, Selangor, Malaysia

**Keywords:** noise-induced hearing loss, self-report, occupational noise, risk, public health

## Abstract

Lifetime noise exposure is generally quantified by self-report. The accuracy of retrospective self-report is limited by respondent recall but is also bound to be influenced by reporting procedures. Such procedures are of variable quality in current measures of lifetime noise exposure, and off-the-shelf instruments are not readily available. The Noise Exposure Structured Interview (NESI) represents an attempt to draw together some of the stronger elements of existing procedures and to provide solutions to their outstanding limitations. Reporting is not restricted to prespecified exposure activities and instead encompasses all activities that the respondent has experienced as noisy (defined based on sound level estimated from vocal effort). Changing exposure habits over time are reported by dividing the lifespan into discrete periods in which exposure habits were approximately stable, with life milestones used to aid recall. Exposure duration, sound level, and use of hearing protection are reported for each life period separately. Simple-to-follow methods are provided for the estimation of free-field sound level, the sound level emitted by personal listening devices, and the attenuation provided by hearing protective equipment. An energy-based means of combining the resulting data is supplied, along with a primarily energy-based method for incorporating firearm-noise exposure. Finally, the NESI acknowledges the need of some users to tailor the procedures; this flexibility is afforded, and reasonable modifications are described. Competency needs of new users are addressed through detailed interview instructions (including troubleshooting tips) and a demonstration video. Limited evaluation data are available, and future efforts at evaluation are proposed.

## Background

**Figure 1.**
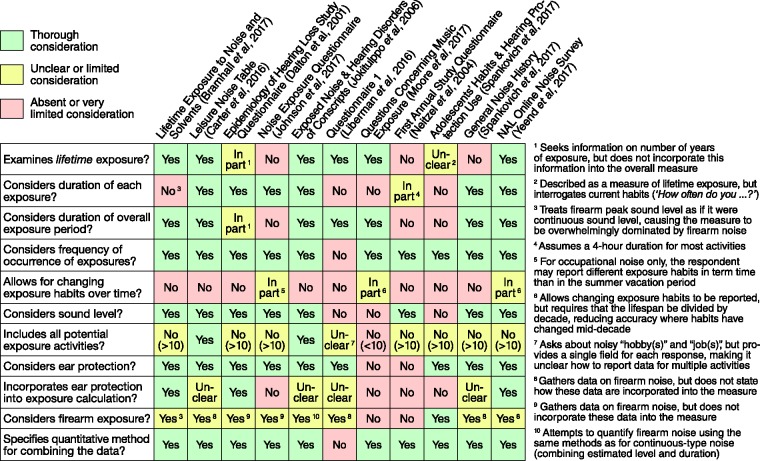
Performance of existing self-report measures of noise exposure.

Research into noise-induced hearing damage has proliferated in recent years. In part, this is attributable to endeavors to determine human physiological and functional correlates of noise-induced cochlear synaptopathy, as demonstrated in animal models (Liberman & Kujawa, 2017). Unlike this animal work, human research predominantly relies on retrospective self-report estimates of cumulative noise exposure. Accuracy of quantification is undoubtedly limited by respondent recall but also by data capture procedures. Numerous methods have been developed independently by different research teams, each to solve the same objective. The first research gap is therefore the lack of standardization of procedure. The second research gap is the comprehensiveness of the estimation procedure itself. Existing procedures tend not to fully consider all of the factors that are important for eliciting an estimate of noise exposure over the lifespan (e.g., Bramhall, Konrad-Martin, McMillan, & Griest, 2017; Carter, Black, Bundy, & Williams, 2016; Dalton et al., 2001; Johnson, Cooper, Stamper, & Chertoff, 2017; Jokitulppo, Toivonen, & Björk, 2006; Liberman, Epstein, Cleveland, Wang, & Maison, 2016; Moore, Zobay, Mackinnon, Whitmer, & Akeroyd, 2017; Neitzel, Seixas, Goldman, & Daniell, 2004; Spankovich, Le Prell, Lobarinas, & Hood, 2017; Yeend, Beach, Sharma, & Dillon, 2017). Figure 1 reports on these factors and summarizes the performance of existing methods. While some of the procedures appear more comprehensive than others, few allow public access to the instrument *per se*. This identifies the third research gap, which is lack of publication of the administrator instructions, record forms, checklists, and calculations of noise units, at least as an “off-the-shelf” solution that can readily be used, in a consistent manner, by researchers elsewhere.

The Noise Exposure Structured Interview (NESI) represents the first effort to go beyond simply describing a procedure for estimating lifetime noise exposure based on self-report, by offering a comprehensive and ready-made solution that we intend as a common standard for the field. This article presents the complete instrument, including a description of the procedure and all supporting materials for self-directed “training” and for administration. The NESI does not claim to contain completely novel elements; indeed, some of its elements are adopted from existing procedures, notably the Noise Exposure and Rating Questionnaire published in a Health and Safety Executive report (Lutman, Davis, & Ferguson, 2008), which was originally developed for the UK National Study of Hearing (A. C. Davis, 1995; Lutman & Spencer, 1991), and utilized in a number of other projects (e.g., Browning, 1986; Smith, Davis, Ferguson, & Lutman, 2000). Rather, the innovation and scientific value lie in the way the procedures are packaged together and integrated with novel elements, yielding an instrument that is comprehensive, clear, and not unduly time-consuming for the administrator.

Methods have been developed in an iterative manner using insights from at least seven coauthors and external colleagues who conducted “beta” testing of preliminary versions. Of the various preliminary versions (see e.g., Prendergast et al., 2017, 2018), those bearing closest resemblance to the current NESI are the versions reported by Guest, Munro, Prendergast, Howe, and Plack (2017) and Dewey et al. (2018), which differ from the NESI in terms of interview instructions and aspects of the supporting documents, but would be unlikely to produce markedly different results. We define the current instrument as “NESI version 1,” in order to explicitly acknowledge the potential for subsequent refinement and revision, as deemed necessary. However, for brevity, the remainder of this article refers to the instrument simply as “the NESI.”

## Concept

The structured interview aims to elicit data on the level and duration of noise exposures over the lifespan, along with usage and attenuation of hearing protection devices (HPDs). The great challenge when collecting such data is that exposure activities and patterns of exposure are unique to the individual and change over time. In addressing these problems, the NESI adopts an approach which is flexible but also highly structured.

Reporting is not restricted to prespecified exposure activities and instead encompasses all activities that the respondent has experienced as noisy (defined based on sound level estimated from vocal effort). Changing exposure habits over time are reported by dividing the lifespan into periods in which exposure habits were approximately stable, with life milestones used to aid recall. Within each life period, standardized methods are used in the estimation of sound level, duration, and attenuation of HPDs. A suggested means of combining these data is provided, based on total energy of exposure, along with a primarily energy-based method for incorporating firearm-noise exposure.

## Methods

### Structure and Documentation

Practical administration of the NESI requires three documents, supplied as Supplementary Material:

- The NESI worksheets (for recording recreational, occupational or educational, and firearm noise exposure; Supplementary Material 1).
- The NESI guidance (overview, instructions, recreational noise examples, speech communication table, personal listening device table, and hearing protection guide; Supplementary Material 2).
- The NESI example calculations (a spreadsheet demonstrating calculation of units of noise exposure; Supplementary Material 3).

Additional background materials are also supplied:

- Further information on the methods for estimating free-field sound level based on vocal effort (Supplementary Material 4).
- Further information on the methods for estimating attenuation of HPDs (Supplementary Material 5).
- Further information on the methods for quantifying firearm noise exposure (Supplementary Material 6).
- A video demonstrating NESI procedures for training and familiarization purposes (available at https://youtu.be/bqgz7-_wmYA).

The methods by which noise exposure data are obtained and combined fall into seven basic categories: (a) identification of exposure activities, (b) segmentation of the lifespan, (c) estimation of exposure duration, (d) estimation of exposure level, (e) consideration of hearing protection, (f) quantification of firearm noise exposure, and (g) calculation of noise exposure units.

### Identification of Exposure Activities

Restricting reporting to prespecified activities is common in measures of noise exposure, but risks underestimating the exposure of respondents who engage in activities that are less common, or less commonly associated with high sound levels. An additional risk is the overreporting of activities which *can* involve high sound levels but do not always do so (e.g., quieter bars and concerts). The NESI follows Lutman et al. (2008) in allowing the respondent to report all noisy (>80 dBA) activities that they have experienced (see also Smith et al., 2000). A “noisy” environment is defined as one in which the respondent would need to raise his or her voice to communicate (at a distance of 4 feet, communicating with a listening partner with normal hearing, with gestures and facial cues available to aid communication).

Although identification of exposure activities is ultimately determined by the respondent’s report, we have elected to provide prompts to expedite this process. Recreational Noise Examples (on p. 8 of Supplementary Material 2) are provided to the respondent early in the interview. These examples were derived from preliminary data from respondents with varying ages, backgrounds, and noise exposures, obtained using measures closely related to the NESI. Listed activities were those reported by 4 or more out of ∼250 respondents. Crucially, this list of examples is not exhaustive, and respondents are explicitly instructed to also report any other activities they perceived as noisy (i.e., requiring a raised voice to communicate). Similarly, they are instructed to ignore any activities that appear on the list but which they did not perceive as noisy.

### Segmentation of the Lifespan

Exposure habits vary across the lifespan. This can be true of not only choice of exposure activities but also frequency of occurrence, sound level, usage of hearing protection, and so on. Reporting of current habits is likely to be unrepresentative of lifetime exposure patterns, especially in older respondents. One solution, utilized by Yeend et al. (2017) and Moore et al. (2017), is to segment the lifespan into decades and assess noise exposure habits in each. However, this framework is likely to compromise accuracy where exposure habits have changed markedly mid-decade, for example, if a respondent attended nightclubs from 18 to 22 (incurring 2 years of exposure in the second decade of life, and two in the third).

A more accurate approach is to segment the lifespan *on the basis of exposure habits*. Hence, the NESI prompts respondents to divide the lifespan into periods in which exposure habits were approximately stable (e.g., time spent as a university student). Patterns of exposure are then recorded for each life period separately, until reporting across the lifespan is complete. Since exposure habits may change for one activity but not others, life periods are identified for each activity separately.

The authors have observed an additional benefit of this approach: life events can be used as points of reference to improve quality of recall, as in the Noise History Calendar (Welch, John, Grynevych, & Thorne, 2011). Hence, the NESI provides fields for recording the timing of each exposure period and advises that any contemporaneous life milestones (e.g., graduation or change of workplace) be noted to assist recall (see Step 5 of the NESI instructions in Supplementary Material 2).

### Estimation of Exposure Duration

To estimate total exposure duration within each life period, the interviewer requires information on typical duration and frequency of occurrence of exposures. Following Lutman et al. (2008), we have elected to express exposure frequency in weeks per year and days per week. Broader subdivisions (e.g., days per month and months per year) are inappropriate for some purposes, such as the reporting of occupational exposure patterns that remain constant from week to week.

However, recording of data in this format is not always straightforward. For example, a respondent might report engaging in an activity “twice a month.” In these cases, it falls to the interviewer to convert these data to fit the NESI framework (e.g., “twice a month” = 24 weeks per year × 1 day per week). The need to perform such conversions is highlighted in Step 7 of the NESI instructions (Supplementary Material 2).

### Estimation of Exposure Level

Three basic approaches to the quantification of sound level are employed in existing self-report measures of noise exposure:

- No consideration of sound level; all exposure activities are weighted equally (e.g., Liberman et al., 2016; Moore et al., 2017).
- Sound level is estimated for each exposure activity using databases of sound level measurements (e.g., Bramhall et al., 2017; Johnson et al., 2017; Yeend et al., 2017).
- Sound level is estimated by the participant, based on communication difficulty (e.g., Guest et al., 2017; Jokitulppo et al., 2006; Keppler, Dhooge, & Vinck, 2015; Lutman et al., 2008).

Method (b) has some advantages, principally in reducing the time taken to complete the measure and in circumventing concerns about the accuracy of respondent estimates. However, we propose that method (c) may be preferable, for the following reasons:

- For some exposure activities, especially those associated with less commonplace occupations, no sound level measurements may be available.
- For activities that *are* included, the listed sound levels may not reflect the full range of levels possible for that activity and may therefore be misleading. For example, sound levels associated with sailing, listed at 45 dBA in the Noise Navigator™ database (Berger, Neitzel, & Kladden, 2015), were estimated to exceed 80 dBA by several preliminary NESI respondents.
- Within a single activity, a very wide range of sound levels is often listed, for example, 67 to 88 dBA for restaurants in the NOISE database (Beach, Gilliver, & Williams, 2013). A means of choosing among them, guided by the respondent, is required.
- Respondents are capable of estimating noise levels with reasonable accuracy, given a loudness rating scale based on communication difficulty (Beach, Williams, & Gilliver, 2012; Ferguson, Tomlinson, Davis, & Lutman, 2018).

Hence, the NESI procedure incorporates respondent-estimated sound level. The Speech Communication Table (Ferguson et al., 2018; Lutman et al., 2008) prompts the respondent to estimate the vocal effort that (s)he would require to communicate in a given environment, at a distance of 4 feet, assuming that the listener is not hearing impaired, is not wearing hearing protective equipment, and may be assisted by gestures and facial cues (see p. 9 of Supplementary Material 2). Note that only the hypothetical listener in this scenario is required to have normal hearing, not the talker (the NESI respondent), who may be hearing impaired. The present version of the table was adapted from that reported by Lutman and colleagues (see Supplementary Material 4). Evaluation data have been obtained for the use of this procedure in estimating occupational noise levels (Ferguson et al., 2018), though not for recreational exposures and not for exposures in the distant past (see Evaluation section of the present article). We recognize that some NESI users may wish to adopt an alternative approach, such as using respondent estimates for only those activities omitted from databases of sound-level measurements. To facilitate this approach, the NESI worksheets (Supplementary Material 1) include extra fields for recording estimates from an alternative source.

Finally, for earphones or headphones used with personal listening devices (PLDs), we have developed the Personal Listening Device Table (p. 10 of Supplementary Material 2): a tool for estimating free-field equivalent output level based on typical volume control setting. Conversion values are based on approximate mean levels measured by Portnuff, Fligor, and Arehart (2011), using a range of devices coupled to stock earphones. These values are also consistent with EU standards governing maximum sound levels of PLDs (British Standards Institution, 2017). Note that the Personal Listening Device Table applies only to PLDs, not to earphones used with other devices (e.g., stereos or personal computers). For such exposures, sound level may be estimated by eliciting comparisons to other activities previously reported by the participant (e.g., “louder than”, “similar loudness to”, or “quieter than” an activity whose sound level has already been estimated).

It is important to note that, although we have attempted to provide sound-level estimation methods for most common noisy activities, omissions remain. For example, for musicians performing at amplified live-music events, sound from in-ear monitors contributes to personal exposure (Federman & Ricketts, 2008), yet levels could not be easily estimated using the NESI (nor, indeed, using any of the procedures reported in Table 1). Hence, caution and common sense must be employed when attempting to quantify the exposure of some music-industry professionals and students.

### Consideration of Hearing Protection

HPDs reduce sound levels in the ear canal but may be worn inconsistently. Hence, to quantify their effects, the NESI examines the approximate proportion of time that HPDs were used, as well as their estimated attenuation. The former is estimated by the respondent; the latter is derived from attenuation ratings published by HPD manufacturers.

To assist the user in estimating the attenuation of HPDs, we have developed the NESI Hearing Protection Guide (pp. 11–12 of Supplementary Material 2). Several possible routes to an estimate are provided, since, in our experience, respondents vary greatly in their recollection of protector type, from vague descriptions of shape through to precise reports of make and model. Pictorial representations of protector types are provided, along with attenuation values for several popular HPDs, and guidance on estimating attenuation based on the product’s single number rating or noise reduction rating.

Supplementary Material 5 provides detailed information on the quantitative methods by which our attenuation estimates are derived, and the reasoning behind these methods.

### Quantification of Firearm Noise Exposure

Over the decades, damage risk criteria have employed a variety of methods for quantifying firearm noise exposure. Early metrics based on peak level and duration have been succeeded by metrics based on the entire temporal waveform (R. R. Davis & Clavier, 2017). Prominent among the latter is A-weighted equivalent continuous 8-hr level (L_Aeq8hr_), which has been recommended by the National Institute for Occupational Safety and Health (Murphy & Kardous, 2012), the American Institute of Biological Sciences (Wightman, Flamme, Campanella, & Luz, 2010), and Defence Research and Development Canada (Nakashima, 2015). One clear benefit of this metric is that it can be easily integrated with energy-based measures of continuous-type noise exposure (Nakashima, 2015).

However, a significant body of research indicates that impulsive noise is more damaging to the auditory system than continuous-type noise of equal energy (e.g., Dunn, Davis, Merry, & Franks, 1991; Hamernik & Qiu, 2001). In the context of damage risk criteria, there is growing support for energy-based metrics that are adjusted for the greater kurtosis (peakedness) of impulsive noise (e.g., R. R. Davis & Clavier, 2017; Murphy & Kardous, 2012). Sounds with greater kurtosis cause greater permanent threshold shift than Gaussian noise of equal energy (R. I. Davis et al., 2012; Hamernik & Qiu, 2001; Hamernik, Qiu, & Davis, 2007). Adjusting noise metrics for kurtosis improves their capacity to predict permanent threshold shift in humans (Goley, Song, & Kim, 2011; Xie et al., 2016; Zhao et al., 2010). The NESI has adopted the kurtosis-corrected metric of Goley et al. (2011):

L'Aeq=LAeq+4.02×log10(β/βG)

where L′_Aeq_ is kurtosis-corrected A-weighted equivalent continuous level, L_Aeq_ is uncorrected A-weighted equivalent continuous level, 4.02 is a constant derived from dose-response data in chinchillas, β is the kurtosis statistic of the noise, and β_G_ is the kurtosis statistic for Gaussian noise (β_G_ = 3).

Incorporation of firearm noise into the NESI can therefore be achieved by combining L_Aeq_ and β, as measured at the shooter’s ear. Flamme, Wong, Liebe, and Lynd (2009) and Meinke et al. (2014) have reported these data for a variety of firearms. More specifically, Flamme et al. report A-weighted equivalent continuous 8-hr level (L_Aeq8hr_): the A-weighted noise level that, if present over an 8-hr period, would contain the same sound energy as the firearm impulse. Due to a markedly bimodal distribution of L_Aeq8hr_, we have elected to dichotomize these weapons into low-caliber (.22 and .17) rifles and all other hand-held firearms (with the exception of air guns, see later). Mean L_Aeq8hr_ for each category has been combined with a kurtosis correction term, yielding kurtosis-corrected A-weighted exposure energy for each category. These values are presented for the NESI user as fractions of a NESI unit of noise exposure, which should be multiplied by the total number of rounds fired.

Exposures to air guns and exposures while wearing hearing protection are disregarded, due to their very low exposure energy. Quantitative justification for this decision is provided in Supplementary Material 6, as are details of all calculations outlined above. Exposure to impulsive noise from sources other than firearms (e.g., artillery and blast noise) is beyond the scope of the NESI.

Finally, it is worth noting that, for the sake of simplicity, NESI procedures for quantification of firearm noise are more rudimentary than those for continuous-type noise in recreational or occupational settings. The firearm noise worksheet (Supplementary Material 1) allows the respondent to estimate the total number of rounds fired in whatever manner they choose. (The field labeled “Additional information to assist recall” may be used to note number of rounds per session, sessions per year, etc.) This contrasts with the more prescriptive approach adopted in the other worksheets. In addition, as stated earlier, firearms are dichotomized, and exposures while wearing hearing protection disregarded. Although preliminary NESI respondents (who were generally UK residents) reported relatively little firearm exposure, we appreciate that other populations may be more highly exposed. Supplementary Material 6 provides guidance on implementing a more fine-grained approach, if required.

### Calculation of Noise Units

The NESI is primarily a procedure for collecting noise exposure data. However, a suggested means of *combining* these data is also provided, based on that of Lutman et al. (2008).

For exposure activities where no hearing protection was worn:

$$\mathrm{Unitsofnoiseexposure}=\frac{Y\times W\times D\times H}{2080}\times 10^{\frac{L-90}{10}}$$

For exposure activities where hearing protection was worn and reduced sound levels to ≤80 dBA:

$$\begin{array}{c} \mathrm{Units}\mathrm{ofnoiseexposure}\\ =\frac{Y\times W\times D\times H}{2080}\times (1-P)\times 10^{\frac{L-90}{10}} \end{array}$$

For exposure activities where hearing protection was worn and did not reduce sound levels to ≤80 dBA:

$$\begin{array}{c} \mathrm{Unitsofnoiseexposure}\\ =\frac{Y\times W\times D\times H}{2080}\times (P\times 10^{\frac{L-A-90}{10}}+(1-P)\times 10^{\frac{L-90}{10}}) \end{array}$$

where

*Y*: years of exposure
*W*: weeks per year of exposure
*D*: days per week of exposure
*H*: hours per day of exposure
*P*: proportion of time that hearing protection was worn (from 0 to 1)
*L*: sound level (dBA)
*A*: attenuation of hearing protection

The resulting measure is linearly related to the total energy of exposure above 80 dBA. One unit is equivalent to one working year (2080 hrs) of exposure to 90 dBA (hence “*L*-90” in the above equations). The reasons for focusing on one working year and 90 dBA are largely historical: the equations were originally devised for the assessment of *occupational* noise exposure, at a time when 90 dBA represented an important legal limit. We have elected not to alter the calculations, so that NESI data may be comparable with data obtained using precursor measures. Firearm noise exposure is incorporated using a primarily energy-based metric (see Step 16 of Supplementary Material 2 and further details in Supplementary Material 6).

To aid investigators new to the NESI, an Excel spreadsheet with example calculations is provided (Supplementary Material 3). It is possible to remove the example data and replace with data from verum NESI respondents, and some users may opt for this approach. However, users are advised to carefully consider alternative ways to store and analyze the data.

## Application and Training

The NESI was developed for use in auditory research, but may have wider application, for example in non-auditory research fields and for clinical purposes. Piloting suggests that completion of the interview takes 10 to 25 min for most respondents, excepting those with extremely extensive or complex noise exposure histories. The instructions (Supplementary Material 2) and demonstration video (https://youtu.be/bqgz7-_wmYA) provide guidance on maintaining interview duration within reasonable limits.

Competency in conducting the NESI requires thorough training and practice, due to the potential for interviewer behavior to influence reporting. To maximize both inter- and intrarater reliability, the user must develop a consistent “script” for each stage of the interview. The precise wording of the script may be chosen by the user but must express the points set out in the NESI instructions and be consistent across participants. We recommend that new users carefully study the worksheets, guidance, and additional background materials (Supplementary Materials 1–6 and video) and also conduct several mock interviews before embarking upon data collection.

We recognize that some users may wish to modify the NESI in order to address specific research questions (e.g., quantifying total duration of exposure above a given level or examining exposure at specific stages of the lifespan). The instructions provide guidance on some reasonable modifications and how they might be implemented (p. 7 of Supplementary Material 2). It would be good practice to disclose any deviations from the principal NESI methods when reporting the resulting data.

## Evaluation

The advent of smart-watches and other technologies may soon allow for continuous, long-term, objective measurement of an individual’s noise exposure. For now, the absence of a gold-standard measure of lifetime noise exposure means that self-report metrics must be evaluated piecemeal.

A component of the NESI, the Speech Communication Table, has been evaluated via dosimetry in 15 workplace settings in which noise levels were greater than or equal to 85 dBA (Ferguson et al., 2018). In this study, 168 participants aged 16 to 25 years estimated noise exposure using a version of the Speech Communication Table and wore personal noise dosimetry badges to objectively measure the noise level in the same nominated occupational tasks. In terms of estimation, methods agreed to within ±3 dB in 56% of cases and within ±6 dB in 91% of cases (Ferguson et al., 2018). Lutman et al. (2008) therefore concluded that, “for group comparisons, noise level estimation from self-reported communication difficulty is appropriate” (p. 57). Note, however, that a limitation of this study is that exposures were purely occupational; recreational exposures might pose different challenges.

**Figure 2.**
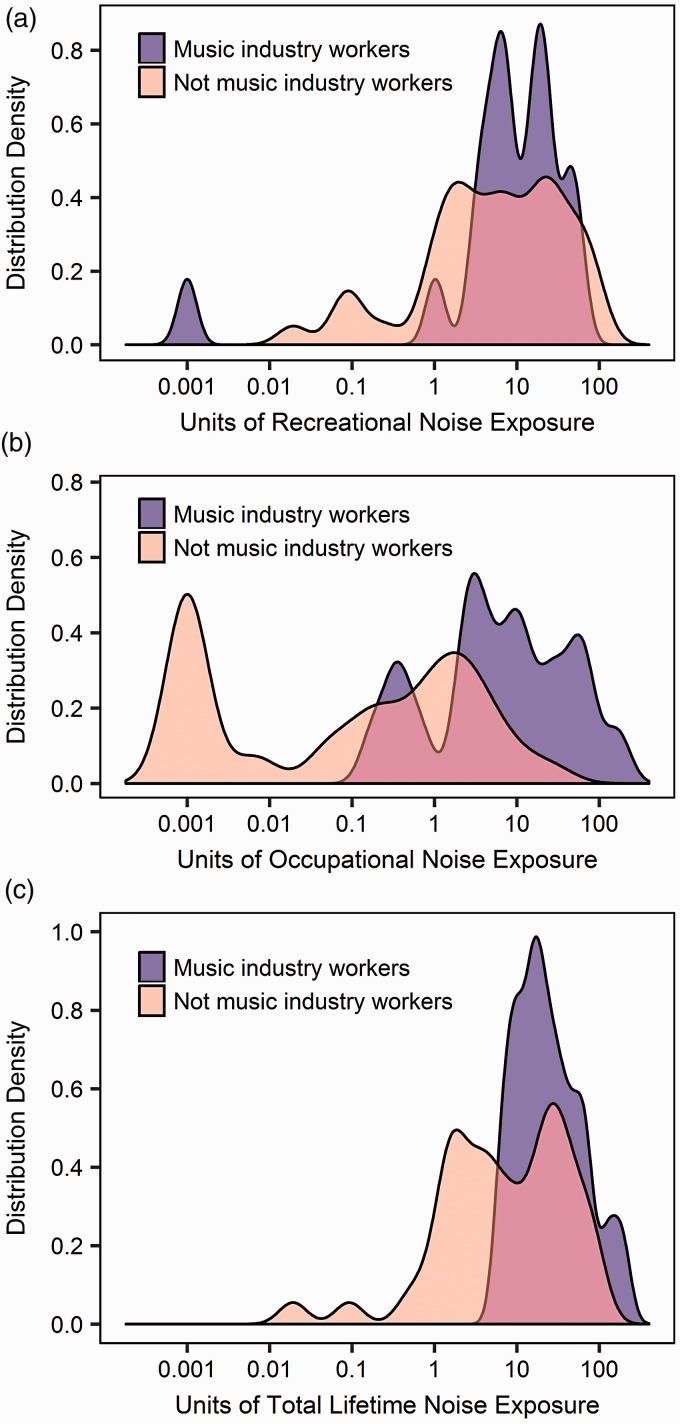
Noise exposure data from a cohort of 62 preliminary NESI respondents, obtained using a beta version of the NESI (Dewey et al., 2018). Nineteen were classed as music-industry workers, the remaining 43 were not. Music-industry workers encompassed professionals, teachers, trainees, and experienced amateurs in the following: musical performance, sound engineering, music production engineering, and disk jockeying. Density plots illustrate the distributions of (a) recreational noise exposure, (b) occupational noise exposure, and (c) total lifetime noise exposure. Note that, to allow plotting on a logarithmic scale, NESI scores of 0 have been adjusted to 0.001.

Feedback from NESI pilot users indicates interviewer confidence in the capacity of the procedures to enhance respondent recall. In preliminary data, exposure to a single activity was often recorded across multiple life periods, suggesting that this framework is of value in capturing changing exposure habits across the lifespan. Preliminary data also demonstrate the NESI’s capacity to distinguish those in noisy professions from other respondents (Figure 2).

Since recreational noise exposure is a major contributor to the lifetime noise dose, a priority for future research should be evaluation of the Speech Communication Table in recreational settings. In addition, evaluation of this procedure for sporadic or erstwhile exposures may be important, since accuracy of recall may diminish over time. It may also be valuable to determine both the intra- and interrater reliability of the NESI.

## Conclusion

Development of the NESI represents an attempt to draw together some of the stronger elements of existing self-report procedures for estimating lifetime noise exposure and to supply novel solutions to their outstanding limitations. Its structure allows the report of an unrestricted range of noisy activities and of changing noise exposure habits over the lifetime, strengthened by a mnemonic approach. Methods are provided for estimating the sound levels of all exposure activities, not only those that are adequately represented in databases of sound-level measurements. Straightforward methods allow the effects of hearing protection to be quantified. An energy-based means of combining the resulting data—including exposure to firearm noise—is supplied. Since some users may wish to deviate according to research needs, the NESI affords the flexibility for reasonable modifications. Training of new users is aided by detailed instructions and a demonstration video. Of course, further evaluation of the NESI instrument is required, and suggestions as to useful modifications in future versions are welcome. Finally, the authors call for the open sharing of data obtained using the NESI, so that the power of large data sets might be harnessed.

## Supplemental Material

Supplemental material1 - Supplemental material for The Noise Exposure Structured Interview (NESI): An Instrument for the Comprehensive Estimation of Lifetime Noise Exposure Click here for additional data file. Supplemental material, Supplemental material1 for The Noise Exposure Structured Interview (NESI): An Instrument for the Comprehensive Estimation of Lifetime Noise Exposure by Hannah Guest, Rebecca S. Dewey, Christopher J. Plack, Samuel Couth, Garreth Prendergast, Warren Bakay and Deborah A. Hall in Trends in Hearing

## Supplemental Material

Supplemental material2 - Supplemental material for The Noise Exposure Structured Interview (NESI): An Instrument for the Comprehensive Estimation of Lifetime Noise Exposure Click here for additional data file. Supplemental material, Supplemental material2 for The Noise Exposure Structured Interview (NESI): An Instrument for the Comprehensive Estimation of Lifetime Noise Exposure by Hannah Guest, Rebecca S. Dewey, Christopher J. Plack, Samuel Couth, Garreth Prendergast, Warren Bakay and Deborah A. Hall in Trends in Hearing

## Supplemental Material

Supplemental material3 - Supplemental material for The Noise Exposure Structured Interview (NESI): An Instrument for the Comprehensive Estimation of Lifetime Noise Exposure Click here for additional data file. Supplemental material, Supplemental material3 for The Noise Exposure Structured Interview (NESI): An Instrument for the Comprehensive Estimation of Lifetime Noise Exposure by Hannah Guest, Rebecca S. Dewey, Christopher J. Plack, Samuel Couth, Garreth Prendergast, Warren Bakay and Deborah A. Hall in Trends in Hearing

## Supplemental Material

Supplemental material4 - Supplemental material for The Noise Exposure Structured Interview (NESI): An Instrument for the Comprehensive Estimation of Lifetime Noise Exposure Click here for additional data file. Supplemental material, Supplemental material4 for The Noise Exposure Structured Interview (NESI): An Instrument for the Comprehensive Estimation of Lifetime Noise Exposure by Hannah Guest, Rebecca S. Dewey, Christopher J. Plack, Samuel Couth, Garreth Prendergast, Warren Bakay and Deborah A. Hall in Trends in Hearing

## Supplemental Material

Supplemental material5 - Supplemental material for The Noise Exposure Structured Interview (NESI): An Instrument for the Comprehensive Estimation of Lifetime Noise Exposure Click here for additional data file. Supplemental material, Supplemental material5 for The Noise Exposure Structured Interview (NESI): An Instrument for the Comprehensive Estimation of Lifetime Noise Exposure by Hannah Guest, Rebecca S. Dewey, Christopher J. Plack, Samuel Couth, Garreth Prendergast, Warren Bakay and Deborah A. Hall in Trends in Hearing

## Supplemental Material

Supplemental material6 - Supplemental material for The Noise Exposure Structured Interview (NESI): An Instrument for the Comprehensive Estimation of Lifetime Noise Exposure Click here for additional data file. Supplemental material, Supplemental material6 for The Noise Exposure Structured Interview (NESI): An Instrument for the Comprehensive Estimation of Lifetime Noise Exposure by Hannah Guest, Rebecca S. Dewey, Christopher J. Plack, Samuel Couth, Garreth Prendergast, Warren Bakay and Deborah A. Hall in Trends in Hearing

## References

[bibr1-2331216518803213] Beach E. F. Gilliver M. Williams W. (2013) The NOISE (Non-Occupational Incidents, Situations and Events) database: A new research tool. Annals of Leisure Research 16(2): 149–159. doi:10.1080/11745398.2013.793157.

[bibr2-2331216518803213] Beach E. F. Williams W. Gilliver M. (2012) The objective-subjective assessment of noise: Young adults can estimate loudness of events and lifestyle noise. International Journal of Audiology 51(6): 444–449. doi:10.3109/14992027.2012.658971. 22380619 10.3109/14992027.2012.658971

[bibr3-2331216518803213] Berger, E. H., Neitzel, R., & Kladden, C. (2015). *Noise Navigator™ sound level database with over 1700 measurement values (version 1.8)*. Retrieved from https://multimedia.3m.com/mws/media/888553O/noise-navigator-sound-level-hearing-protection-database.pdf.

[bibr4-2331216518803213] Bramhall N. F. Konrad-Martin D. McMillan G. P. Griest S. E. (2017) Auditory brainstem response altered in humans with noise exposure despite normal outer hair cell function. Ear and Hearing 38(1): e1–e12. doi:10.1097/AUD.0000000000000370. 27992391 10.1097/AUD.0000000000000370 PMC5313078

[bibr5-2331216518803213] British Standards Institution. (2017). BS EN 60065:2014+A11:2017: Audio, video and similar electronic apparatus—Safety requirements. https://shop.bsigroup.com/ProductDetail/?pid=000000000030338899.

[bibr6-2331216518803213] Browning G. G. (1986) Clinical otology and audiology, 1st ed Oxford, England: Butterworth-Heinemann.

[bibr7-2331216518803213] Carter L. Black D. Bundy A. Williams W. (2016) An estimation of the whole-of-life noise exposure of adolescent and young adult Australians with hearing impairment. Journal of the American Academy of Audiology 27(9): 750–763. doi:10.3766/jaaa.15100. 27718351 10.3766/jaaa.15100

[bibr8-2331216518803213] Dalton D. S. Cruickshanks K. J. Wiley T. L. Klein B. E. Klein R. Tweed T. S. (2001) Association of leisure-time noise exposure and hearing loss. Audiology 40(1): 1–9. doi:10.3109/00206090109073095. 11296936

[bibr9-2331216518803213] Davis A. C. (1995) Hearing in adults, London, England: Whurr.

[bibr10-2331216518803213] Davis R. I. Qiu W. Heyer N. J. Zhao Y. Yang M. Q. Li N. Yao D. (2012) The use of the kurtosis metric in the evaluation of occupational hearing loss in workers in China: Implications for hearing risk assessment. Noise & Health 14(61): 330, doi:10.4103/1463-1741.104903. 23257587 10.4103/1463-1741.104903

[bibr11-2331216518803213] Davis R. R. Clavier O. (2017) Impulsive noise: A brief review. Hearing Research 349: 34–36. doi:10.1016/j.heares.2016.10.020. 27989948 10.1016/j.heares.2016.10.020

[bibr12-2331216518803213] Dewey R. S. Hall D. A. Guest H. Prendergast G. Plack C. J. Francis S. T. (2018) The physiological bases of hidden noise-induced hearing loss: Protocol for a functional neuroimaging study. JMIR Research Protocols 7(3): e79, doi:10.2196/resprot.9095. 29523503 10.2196/resprot.9095 PMC5866298

[bibr13-2331216518803213] Dunn D. E. Davis R. R. Merry C. J. Franks J. R. (1991) Hearing loss in the chinchilla from impact and continuous noise exposure. The Journal of the Acoustical Society of America 90(4): 1979–1985. doi:10.1121/1.401677. 1669963 10.1121/1.401677

[bibr14-2331216518803213] Federman J. Ricketts T. (2008) Preferred and minimum acceptable listening levels for musicians while using floor and in-ear monitors. Journal of Speech, Language, and Hearing Research 51(1): 147–159. doi:10.1044/1092-4388(2008/011). 10.1044/1092-4388(2008/011) 18230862

[bibr15-2331216518803213] Ferguson, M. A., Tomlinson, K. B., Davis, A. C., & Lutman, M. E. (2018). A simple method to estimate noise levels in the workplace based on self-reported speech communication effort in noise. *International Journal of Audiology*. 10.1080/14992027.2019.1600203 31012769

[bibr16-2331216518803213] Flamme G. A. Wong A. Liebe K. Lynd J. (2009) Estimates of auditory risk from outdoor impulse noise. II: Civilian firearms. Noise & Health 11(45): 231–242. doi:10.4103/1463-1741.56217. 19805933 10.4103/1463-1741.56217

[bibr17-2331216518803213] Goley G. S. Song W. J. Kim J. H. (2011) Kurtosis corrected sound pressure level as a noise metric for risk assessment of occupational noises. The Journal of the Acoustical Society of America 129(3): 1475–1481. doi:10.1121/1.3533691. 21428511 10.1121/1.3533691 PMC3188614

[bibr18-2331216518803213] Guest H. Munro K. J. Prendergast G. Howe S. Plack C. J. (2017) Tinnitus with a normal audiogram: Relation to noise exposure but no evidence for cochlear synaptopathy. Hearing Research 344: 265–274. doi:10.1016/j.heares.2016.12.002. 27964937 10.1016/j.heares.2016.12.002 PMC5256478

[bibr19-2331216518803213] Hamernik R. P. Qiu W. (2001) Energy-independent factors influencing noise-induced hearing loss in the chinchilla model. The Journal of the Acoustical Society of America 110(6): 3163–3168. doi:10.1121/1.1414707. 11785817 10.1121/1.1414707

[bibr20-2331216518803213] Hamernik R. P. Qiu W. Davis B. (2007) Hearing loss from interrupted, intermittent, and time varying non-Gaussian noise exposure: The applicability of the equal energy hypothesis. The Journal of the Acoustical Society of America 122(4): 2245–2254. doi:10.1121/1.2775160. 17902860 10.1121/1.2775160

[bibr21-2331216518803213] Johnson T. A. Cooper S. Stamper G. C. Chertoff M. (2017) Noise Exposure Questionnaire (NEQ): A tool for quantifying annual noise exposure. Journal of the American Academy of Audiology 28(1): 14–35. doi:10.3766/jaaa.15070. 28054909 10.3766/jaaa.15070 PMC5304605

[bibr22-2331216518803213] Jokitulppo J. Toivonen M. Björk E. (2006) Estimated leisure-time noise exposure, hearing thresholds, and hearing symptoms of Finnish conscripts. Military Medicine 171(2): 112–116. 16578978 10.7205/milmed.171.2.112

[bibr23-2331216518803213] Keppler H. Dhooge I. Vinck B. (2015) Hearing in young adults. Part II: The effects of recreational noise exposure. Noise & Health 17(78): 245, doi:10.4103/1463-1741.165026. 26356366 10.4103/1463-1741.165026 PMC4900507

[bibr124-2331216518803213] Liberman, M. C., Epstein, M. J., Cleveland, S. S., Wang, H., & Maison, S. F. (2016). Toward a differential diagnosis of hidden hearing loss in humans. *PLOS ONE*, *11*(9), e0162726. doi:10.1371/journal.pone.0162726. 10.1371/journal.pone.0162726 PMC5019483 27618300

[bibr24-2331216518803213] Liberman M. C. Kujawa S. G. (2017) Cochlear synaptopathy in acquired sensorineural hearing loss: Manifestations and mechanisms. Hearing Research 349: 138–147. doi:10.1016/j.heares.2017.01.003. 28087419 10.1016/j.heares.2017.01.003 PMC5438769

[bibr25-2331216518803213] Lutman, M. E., Davis, A. C., & Ferguson, M. A. (2008). *Epidemiological evidence for the effectiveness of the noise at work regulations* (Research report no. RR669). Sudbury, England: Health and Safety Executive.

[bibr26-2331216518803213] Lutman M. E. Spencer H. S. (1991) Occupational noise and demographic factors in hearing. Acta Oto-Laryngologica Supplementum 476: 74–84. 10.3109/00016489109127258 2087983

[bibr27-2331216518803213] Meinke D. K. Murphy W. J. Finan D. S. Lankford J. E. Flamme G. A. Stewart M. Jerome T. W. (2014) Auditory risk estimates for youth target shooting. International Journal of Audiology 53(Suppl 2): S16–S25. doi:10.3109/14992027.2013.865845. 24564688 10.3109/14992027.2013.865845 PMC4659434

[bibr28-2331216518803213] Moore D. R. Zobay O. Mackinnon R. C. Whitmer W. M. Akeroyd M. A. (2017) Lifetime leisure music exposure associated with increased frequency of tinnitus. Hearing Research 347: 18–27. doi:10.1016/j.heares.2016.10.030. 27825859 10.1016/j.heares.2016.10.030 PMC5417322

[bibr29-2331216518803213] Murphy, W. J., & Kardous, C. A. (2012). *A case for using A-weighted equivalent energy as a damage risk criterion* (In-depth survey report for the Engineering and Physical Hazards Branch report no. 350–11a). Cincinnati, OH: National Institute for Occupational Safety and Health.

[bibr30-2331216518803213] Nakashima, A. (2015). *A comparison of metrics for impulse noise exposure—Analysis of noise data from small calibre weapons* (Scientific report no. DRDC-RDDC-2015-R243). Toronto, Canada: Defence Research and Development Canada.

[bibr31-2331216518803213] Neitzel R. Seixas N. Goldman B. Daniell W. (2004) Contributions of non-occupational activities to total noise exposure of construction workers. The Annals of Occupational Hygiene 48(5): 463–473. doi:10.1093/annhyg/meh041. 15242844 10.1093/annhyg/meh041

[bibr32-2331216518803213] Portnuff C. D. Fligor B. J. Arehart K. H. (2011) Teenage use of portable listening devices: A hazard to hearing? Journal of the American Academy of Audiology 22(10): 663–677. doi:10.3766/jaaa.22.10.5. 22212766 10.3766/jaaa.22.10.5

[bibr33-2331216518803213] Prendergast G. Guest H. Munro K. J. Kluk K. Léger A. Hall D. A. Plack C. J. (2017) Effects of noise exposure on young adults with normal audiograms I: Electrophysiology. Hearing Research 344: 68–81. doi:10.1016/j.heares.2016.10.028. 27816499 10.1016/j.heares.2016.10.028 PMC5256477

[bibr34-2331216518803213] Prendergast G. Tu W. Guest H. Millman R. E. Kluk K. Couth S. Plack C. J. (2018) Supra-threshold auditory brainstem response amplitudes in humans: Test-retest reliability, electrode montage and noise exposure. Hearing Research 364: 38–47. doi:10.1016/j.heares.2018.04.002. 29685616 10.1016/j.heares.2018.04.002 PMC5993871

[bibr35-2331216518803213] Smith P. A. Davis A. Ferguson M. Lutman M. E. (2000) The prevalence and type of social noise exposure in young adults in England. Noise & Health 2(6): 41. 12689478

[bibr36-2331216518803213] Spankovich C. Le Prell C. G. Lobarinas E. Hood L. J. (2017) Noise history and auditory function in young adults with and without type 1 diabetes mellitus. Ear and Hearing 38(6): 724–735. doi:10.1097/AUD.0000000000000457. 28678080 10.1097/AUD.0000000000000457

[bibr37-2331216518803213] Welch, D., John, G., Grynevych, A., & Thorne, P. (2011). *Assessment of life course noise exposure*. Presented at the 10th International Congress on Noise as a Public Health Problem, London, UK.

[bibr38-2331216518803213] Wightman, F. L., Flamme, G. A., Campanella, A. J., & Luz, G. A. (2010). *Peer review of injury prevention and reduction—Research task area: Impulse noise injury models* (Peer-review report). Reston, VA: American Institute of Biological Sciences.

[bibr39-2331216518803213] Xie H.-W. Qiu W. Heyer N. J. Zhang M.-B. Zhang P. Zhao Y.-M. Hamernik R. P. (2016) The use of the kurtosis-adjusted cumulative noise exposure metric in evaluating the hearing loss risk for complex noise. Ear and Hearing 37(3): 312–323. doi:10.1097/AUD.0000000000000251. 26671317 10.1097/AUD.0000000000000251 PMC4844558

[bibr40-2331216518803213] Yeend I. Beach E. F. Sharma M. Dillon H. (2017) The effects of noise exposure and musical training on suprathreshold auditory processing and speech perception in noise. Hearing Research 353: 224–236. doi:10.1016/j.heares.2017.07.006. 28780178 10.1016/j.heares.2017.07.006

[bibr41-2331216518803213] Zhao Y.-M. Qiu W. Zeng L. Chen S.-S. Cheng X.-R. Davis R. I. Hamernik R. P. (2010) Application of the kurtosis statistic to the evaluation of the risk of hearing loss in workers exposed to high-level complex noise. Ear and Hearing 31(4): 527–532. doi:10.1097/AUD.0b013e3181d94e68. 20588120 10.1097/AUD.0b013e3181d94e68

