# Supplementary material for: The Noise Exposure Structured Interview (NESI): An Instrument for the Comprehensive Estimation of Lifetime Noise Exposure
Source: Trends Hear. 2018 Oct 8;22:2331216518803213. doi: 10.1177/2331216518803213 (PMC6176535; doi:10.1177/2331216518803213)
Supplement: Supplemental material1 - Supplemental material for The Noise Exposure Structured Interview (NESI): An Instrument for the Comprehensive Estimation of Lifetime Noise Exposure [file Supplemental_material1.pdf]

NESI THE NOISE EXPOSURE STRUCTURED INTERVIEW

Category A: Recreational noise exposure

ID: **Date:**

Exposure activity									
Timing of exposure period (e.g. age at start and age at end)									
Exposure duration	Years								
	Weeks per year								
	Days per week								
	Hours per day								
Exposure level	Basis for estimate								
	Estimated level (dBA)								
Optional: Exposure level (alternate estimation method)	Basis for estimate								
	Estimated level (dBA)								
Use of hearing protection	Proportion of time worn (between 0 and 1)								
	Notes on type and attenuation of protector								
	Estimated attenuation (dB)								

NESI THE NOISE EXPOSURE STRUCTURED INTERVIEW

Category B: Occupational and educational noise exposure

ID: **Date:**

Exposure activity									
Timing of exposure period (e.g. age at start and age at end)									
Exposure duration	Years								
	Weeks per year								
	Days per week								
	Hours per day								
Exposure level	Basis for estimate								
	Estimated level (dBA)								
Optional: Exposure level (alternate estimation method)	Basis for estimate								
	Estimated level (dBA)								
Use of hearing protection	Proportion of time worn (between 0 and 1)								
	Notes on type and attenuation of protector								
	Estimated attenuation (dB)								

NESI THE NOISE EXPOSURE STRUCTURED INTERVIEW

Category C: Firearm noise exposure

ID: **Date:**

Type of firearm								
Additional information to assist recall								
Total number of rounds fired without hearing protection								

Summary information

	Category A: Recreational noise	Category B: Occupational and educational noise	Category C: Firearm noise	Total
Number of worksheets used				
Person conducting interview				
