# Supplementary material for: The Noise Exposure Structured Interview (NESI): An Instrument for the Comprehensive Estimation of Lifetime Noise Exposure
Source: Trends Hear. 2018 Oct 8;22:2331216518803213. doi: 10.1177/2331216518803213 (PMC6176535; doi:10.1177/2331216518803213)
Supplement: Supplemental material2 - Supplemental material for The Noise Exposure Structured Interview (NESI): An Instrument for the Comprehensive Estimation of Lifetime Noise Exposure [file Supplemental_material2.pdf]

**Introduction**	Outline how the NESI works<br><br>Explain which activities should be reported
**Recreational noise exposure**	Identify exposure activities<br><br>For each activity, divide the lifespan into periods in which exposure habits were approximately stable<br><br>For each life period…<br>• Estimate ***duration of exposure***<br>• Estimate ***level of exposure***<br>• Record use and type of ***hearing protection***
**Occupational and educational noise exposure**	Identify exposure activities<br><br>For each activity, divide the lifespan into periods in which exposure habits were approximately stable<br><br>For each life period…<br>• Estimate ***duration of exposure***<br>• Estimate ***level of exposure***<br>• Record use and type of ***hearing protection***
**Firearm noise exposure**	For any exposures without hearing protection...<br>• Record ***type of firearm***<br>• Estimate ***number of rounds fired***
**Analysis**	For each activity and life period, combine the above data into units of noise exposure<br><br>Add them together, yielding ***total units of lifetime noise exposure***

Use the **recreational noise examples** (page 8) to aid identification of noisy recreational activities

Use the **speech communication table** (page 9) to estimate the sound level of free-field exposures

Use the **personal listening device table** (page 10) to estimate the sound level from earphones/headphones

Use the **hearing protection guide** (pages 11-12) to estimate the attenuation of hearing protectors

Use the **worksheets** to record the respondent's noise exposure information

Steps 1 to 2: Introducing the NESI

1. Outline the purpose of the interview: to identify activities involving high sound levels and estimate the duration and level of exposure for each.

Suggested script:
The purpose of this interview is to estimate your lifetime noise exposure. We will identify activites that have caused you to be in noisy situations, then estimate how long you spent in those situations and how noisy they were.

2. Define the activities that should be reported: those with an estimated sound level exceeding 80 dBA.

For free-field exposures, this is based on estimated vocal effort required to communicate (see **Speech Communication Table**). Instruct the respondent to report situations requiring a "raised voice" at a communication distance of 4 feet (1.2 m).

For personal listening devices, instruct the respondent to report listening at volume control settings above 70% of maximum volume (see **Personal Listening Device Table**).

Suggested script:
I'll explain what I mean by a "noisy" situation: It's the kind of situation where, if you and I were 4 feet apart, you would have to raise your voice to communicate with me. [Interviewer gestures to indicate a distance of 4 feet.] *Assume that I have normal hearing, that I am not wearing ear plugs or ear muffs, and that we are able to see each other's faces and gestures clearly.*

Situations that are at least this noisy are the ones you should report. Quieter situations, which don't cause you to raise you voice, can be ignored.

Finally, there's one other kind of noisy activity we'll look at, and that's listening through earphones or headphones with the volume control set quite high, above 70% of maximum volume.

Tip:

It is sometimes necessary to emphasise that the communication scenario used to estimate free-field noise levels is *hypothetical*.

The respondent should imagine attempting to communicate with a listener without hearing protection, even if this scenario would be unlikely to occur in the environment in question.

Steps 3 to 11: Estimating recreational, occupational, and educational noise exposure

Record recreational exposures (Category A) first, followed by occupational and educational exposures (Category B). Interview methods are similar for the two categories.

3. Prompt the respondent to identify exposure activities. For Category A, these should be noisy activities that occurred recreationally; for Category B, noisy activities that occurred in the course of work or study.

Provide pen and paper and allow the respondent a few minutes alone to note exposure activities. For Category A, also provide the **Recreational Noise Examples**.

The respondent should use these notes later, to aid their recollection. The notes will not be analysed. They may add to the notes at any time.

Tip:

In Category A, what appears initially to be a single "activity" may be divisible into more specific activities with differing sound levels and/or use of hearing protection (e.g. "metal bands" and "folk bands"). If the respondent is able to recall information about these exposures separately, then record them as separate activities.

Similarly, in Category B, make sure to distinguish between a job and an activity. One job may involve multiple activities, only some of which may be noisy.

4. Next, you will examine the identified exposure activities, one after another, estimating sound level, duration, and use of hearing protection for each one.

Encourage the respondent to report their most significant sources of noise exposure early in the interview.

Suggested script:

Now let's look at one activity in detail. Which do you think has contributed most to your overall noise exposure?

5. Outline the process for recording exposure patterns across time: dividing the lifespan into periods in which exposure habits were approximately stable.

A single activity may be reported across multiple life periods if exposure habits have significantly altered.

If not, or if the respondent can't recall, then they should report *average* exposure habits for the entire period of exposure (e.g. age 18 to 45).

Suggested script:

Now we need to estimate how long you have spent [engaging in the activity].

As a first step, I'll ask you to think of a period of your life when your habits were fairly stable: a number of years where there weren't major changes in how often you [engaged in the activity], or how noisy it was, or how long the noise lasted. You might need to divide your life into a several periods. For example, if you [engaged in the activity] very frequently from 16 to 22 and less frequently from 22 to 30, we would look at each period separately.

6. Prompt the respondent to identify a life period. Note the timing (e.g. "age 22 to 30") in the second row of the worksheet. This information may be useful later in the interview, when checking for "gaps" in the exposure record (life periods which involved exposure, but haven't yet been recorded).

Record the duration of this period in years. (This doesn't have to be a whole number.)

Suggested script:

So, when it comes to [the present activity], which period of your life should we look at first?

Tip:

Encourage the participant to make use of relevant life milestones to structure their recollection (e.g. change of workplace, graduation from university).

7. For the activity and life period identified above, prompt the respondent to estimate weeks per year and days per week of exposure. (These don't have to be whole numbers.)

Tip:

Participants sometimes find it easier to express this information in alternative terms (e.g. days per month or days per year). The interviewer should allow this, then convert the information into weeks per year and days per week.

Example: "Twice a month for 3 years" = 3 years × 24 weeks/year × 1 day/week

8. Prompt the respondent to estimate average hours per day of exposure. (This doesn't have to be a whole number.)

9. Prompt the respondent to estimate the typical sound level associated with the activity.

Standard NESI procedure is to use the **Speech Communication Table** (for free-field exposures) or **Personal Listening Device Table** (for headphones/earphones attached to personal listening devices).

Record the estimated sound level. Also record the information that provided the basis for this estimate (e.g. "shout from 2 feet" from the Speech Communication Table).

It is possible to obtain sound-level estimates from alternative sources. (See "Departing from NESI standard procedure" on page 7 of this guide.) Such estimates may be recorded in the grey-shaded fields on the NESI worksheets, which should otherwise be left blank.

Suggested script for free-field exposures:

I'd like you to estimate how noisy it was when you [engaged in the activity] by answering this question: If you and I were 4 feet apart in that situation, which of the following would you need to do to communicate with me? [The interviewer presents the six options from the **Speech Communication Table**.] *Assume that I have normal hearing, that I am not wearing ear plugs or ear muffs, and that we can see one another's faces and gestures clearly.*

Tip:

The **Personal Listening Device Table** applies only to portable devices such as personal music players and phones, not to stereos, PCs, or sound recording equipment. For such exposures, sound level may be estimated by drawing comparisons to other activities previously reported by the participant (e.g. "louder than," "similar loudness to," or "quieter than" an activity whose sound level has already been estimated).

10. For free-field exposures, obtain information on use of hearing protection: protector type, estimated attenuation, and proportion of time worn (ranging from 0 to 1).

Use the **Hearing Protection Guide** to help identify protector type and estimate attenuation.

Tip:

The participant may express the proportion of time that hearing protection was worn as a percentage. Convert from percentage to proportion by dividing by 100 (e.g. 70% ⟶ 0.7).

If hearing protection was never worn, record proportion of time worn as 0.

11. One worksheet column is now complete. Repeat steps 7 to 10 for each additional period of the respondent's life which involved the present activity. Then repeat for each additional exposure activity.

If you run out of columns, attach extra worksheets as needed.

Tip:

Be prepared to split the content of a column into multiple columns, if necessary. A respondent may initially report consistent exposure habits for a given activity, only for further questioning to reveal otherwise (e.g. changes in frequency of exposure, level of exposure, or use of hearing protection).

Steps 12 to 13: Estimating firearm noise exposure

12. Determine whether the respondent has ever used a rifle, shotgun, or handgun ***without hearing protection***. (Exposures while wearing hearing protection should be ignored. Exposure to air guns should be ignored.)

It is possible to implement a modified version of the NESI which incorporates firearm exposure while wearing hearing protection. (See "Departing from NESI standard procedure" on page 7 of this guide.)

Tip:

The NESI quantifies exposure to firearms only. Exposure to heavier weapons and blast noise is beyond the scope of the measure.

13. For exposures ***without hearing protection***, record type of firearm and approximate number of rounds fired on the Category C worksheet. Low-calibre (.22 and .17) rifles are assigned fewer units of noise exposure than other firearms, so make sure to establish whether this type of firearm was used.

Tip:

Complete as many columns as are needed to capture the respondent's exposure history. More than one column may be completed per firearm type. Notes may be made in the "Additional information..." field to assist in estimating the total number of rounds fired (e.g. rounds per session and sessions per year). The contents of this field will not be analysed.

Steps 14 to 17: Recording and analysis

14. Complete at least one worksheet for each of the three categories. Even if the respondent reports no noise exposure in a given category, fill in the fields at the top right corner and retain the worksheet with the others.

If a respondent requires more than one worksheet for a given category, attach extra worksheets as required.

Tip:

Completion of the NESI takes 10-25 minutes for most respondents, excepting those with extensive or complex noise histories.

If an interview risks overrunning the available time, reinstruct the respondent to reduce the precision of their reporting. For example, rather than describing three life periods with slightly differing exposure habits, the respondent may report average exposure habits over one long period.

Do not adopt this approach with the ***earlier*** exposure activities in each category (i.e. those that contributed most to overall exposure). Instead, reserve this approach for later in the interview, when dealing with more minor sources of noise exposure.

The priority is to ensure that the interview is not cut off, and that all activities are reported across the full lifespan.

15. Calculate units of recreational, occupational, and educational noise exposure.

Use the following formulae to generate noise units for each completed column on worksheets A and B.

The **NESI example calculations** spreadsheet shows how these formula are applied, using example NESI data.

Calculation of noise units in Categories A and B:

If hearing protection was not worn...

$$\textit{Units of noise exposure} = \frac{Y \times W \times D \times H}{2080} \times 10^{\frac{L-90}{10}}$$

If hearing protection was worn, and reduced the sound level to <80 dBA...

$$\textit{Units of noise exposure} = \frac{Y \times W \times D \times H}{2080} \times (1-P) \times 10^{\frac{L-90}{10}}$$

If hearing protection was worn, but did not reduce the sound level to <80 dBA...

$$\textit{Units of noise exposure} = \frac{Y \times W \times D \times H}{2080} \times \left(P \times 10^{\frac{L-A-90}{10}} + (1-P) \times 10^{\frac{L-90}{10}}\right)$$

where *Y = years of exposure*
W = weeks per year of exposure
D = days per week of exposure
H = hours per day of exposure
L = level (dBA)
A = attenuation of hearing protection (dB)
P = proportion of time that hearing protection was worn (0 to 1)

16. Calculate units of firearm noise exposure.

Use the following formula to generate noise units for each of the completed columns on worksheet C.

The **NESI example calculations** spreadsheet shows how this formula is applied, using example NESI data.

Calculation of noise units in Category C:

$$\textit{Units of noise exposure} = \frac{R}{500} + \frac{R_{low}}{16000}$$

where *R = number of rounds fired from shotguns, handguns, and rifles, excluding low-calibre (.22 and .17) rifles and air guns*
R_{low} *= number of rounds fired from low-calibre (.22 and .17) rifles*

17. Add the units from all columns to yield total units of lifetime noise exposure, a measure linearly related to total energy of exposure above 80 dBA. One unit equates to one working year (2080 hours) of exposure to 90 dBA.

Alternate units may be generated. See “Departing from NESI standard procedure” on page 7 of this guide.

Departing from NESI standard procedure

Recommended procedure for administering the NESI is fully specified by:

- Steps 1 to 17 of the above guide
- The Recreational Noise Examples
- The Speech Communication Table
- The Personal Listening Device Table
- The Hearing Protection Guide

However, it is recognised that some users of the NESI may wish to modify elements of the procedure. **Any such modifications must be disclosed when reporting data obtained using the NESI**. Some anticipated modifications are outlined below, along with guidance on their implementation.

Modification 1: Estimating exposure level

NESI standard procedure involves estimating sound level using the Speech Communication Table and the Personal Listening Device Table.

It is possible to obtain sound-level estimates from other sources, e.g. databases of sound level measurements. The NESI worksheets include fields for recording an alternative estimate, so that either may be used in analysis.

Modification 2: Altering the criterion sound level for exposure activities

The NESI records all exposures with an estimated sound level >80 dBA, and the standard analysis generates noise units linearly related to total energy of exposure above this level. Some NESI users may wish to apply a higher criterion level in the course of analysis (e.g. analysing only exposures >100 dBA). Such users should implement this modification by amending the structure in which they store and process NESI data, so that inclusion of an activity in the overall NESI score is conditional upon sound level.

Modification 3: Generating units of firearm noise exposure

Standard NESI procedure assigns 1/16000 noise units to each round from a .22 or .17 rifle and 1/500 units to a round from any other handheld firearm, considering only those exposures incurred without hearing protection. These values are based principally on the approximate energy of such exposures, with an adjustment for the kurtosis of the sound waveform.

Alternate analysis methods are possible, e.g. assigning different values to different types of firearm, and/or incorporating exposures incurred with hearing protection. Users considering such modifications should refer to the NESI dissemination paper, which details the basis for the standard NESI weighting of firearm noise and outlines some possible modifications.

Modification 4: Generating total units of lifetime noise exposure

Standard NESI units of lifetime noise exposure are linearly related to the total energy of exposure above 80 dBA. One unit is equivalent to one working year (2080 hours) of exposure at 90 dBA.

Some NESI users may wish to generate an alternative measure, e.g. log energy of exposure, or total duration of exposure exceeding a criterion sound level, or total units of occupational noise exposure. Such users should implement this modification by amending the structure in which they store and process NESI data.

Modification 5: Examining noise exposure during specific periods

Standard NESI output is a measure of cumulative lifetime noise exposure.

Some users may wish instead to examine the timing of exposures (e.g. focusing on exposures during childhood, or exposures preceding the development of hearing deficits). In this case, the interviewer must ensure that the “Timing of exposure period” field is always completed, and should amend the structure in which they store and process NESI data, so that inclusion of an exposure in the overall NESI score is conditional upon timing.

- Your interviewer has asked you to note any noisy activities you have experienced. Listed below are some common noisy activities, which may help to prompt your memory.
- **Remember what is meant by "noisy"**: situations causing you to **raise your voice** to communicate at a distance of 4 feet (1.2 m).
- **Do not restrict yourself to the activities on this list**. Also note any other noisy activities you have experienced.
- **Only note activities that you have found to be noisy**. If you have experienced an activity on this list but did not find it noisy, then **ignore it**.

Live music

Examples:
- Concerts
- Festivals

Nightlife

Examples:
- Nightclubs
- Bars
- Pubs

Making music

Examples:
- Playing/singing in a group
- Playing/DJing/singing solo

Listening through earphones or headphones

DIY noise

Examples:
- Power tools
- Powered gardening tools

Engine noise

Examples:
- Motorbikes
- Motorsports
- Motorboats

Sport-related noise

Examples:
- Sports matches
- Sailing

Cinema

Vocal effort required	*Estimated level*
**Talk normally** from 4 feet (1.2 m)	≤80 dBA
**Raise voice** from 4 feet (1.2 m)	87 dBA
**Talk loudly** from 4 feet (1.2 m)	90 dBA
**Talk very loudly** from 4 feet (1.2 m)	93 dBA
**Shout** from 4 feet (1.2 m)	99 dBA
**Shout from 2 feet (0.6 m)**	105 dBA
**Shout in listener's ear**	110 dBA

A guide for estimating unknown noise levels (of a continuous type) based on speech communication difficulty.

Approximate communication-limiting noise levels are based on the scenario of one person communicating with another in an environment that they are both used to, assuming that the listener is not hearing impaired, is not wearing hearing protection, and may be assisted to some extent by gestures and facial cues.

A guide for estimating the free-field equivalent output levels of earphones or headphones coupled to personal listening devices (e.g. phones and music players), based on the respondent's typical volume control setting.

Volume control setting	Estimated level
<70% of maximum	<80 dBA
70% of maximum	82 dBA
80% of maximum	88 dBA
90% of maximum	94 dBA
Maximum volume	100 dBA

Output-level warning messages in European devices

Many personal listening devices sold in the European Union from February 2013 have settings designed to encourage listening levels below 85 dBA*. When sound levels reach 85 dBA, the listener is presented with a visual or audible warning message which they must acknowledge in order to access the upper portion of the volume control range.

Note that "maximum volume" in the above table does ***not*** refer to the sound level that elicits this warning message. "Maximum volume" refers to the true upper limit of the volume control range, accessed by acknowledging the message and further increasing the sound level.

If a respondent has encountered such warning messages, then their recollection of this phenomenon can sometimes assist in estimating sound level (for example, if they consistently chose not to exceed the 85 dB warning level).

**BS EN 60065:2014+A11:2017: Audio, video and similar electronic apparatus – Safety requirements.*

NESI THE NOISE EXPOSURE STRUCTURED INTERVIEW

Hearing Protection Guide

A tool for estimating the attenuation of hearing protectors.

NESI "estimated attenuation" is derived from attenuation ratings reported by manufacturers: either the **Single Number Rating (SNR)**, used primarily in Europe, or the **Noise Reduction Rating (NRR)**, used in the US and elsewhere.

- **If the specific model of protector is listed in this guide**, simply read its estimated attenuation from the table
- **If the specific model is not listed, but its SNR or NRR is known**, calculate its estimated attenuation using the final table
- **For all other hearing protectors**, estimate attenuation based on the ***type*** of hearing protector

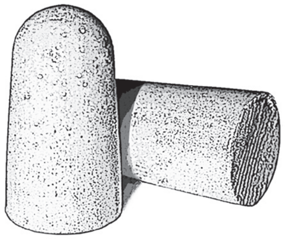

Formable ear plugs

	Estimated attenuation
3M E-A-R Classic (SNR 28)	24 dB
Howard Leight Laser Lite (SNR 35)	31 dB
Moldex SparkPlugs (SNR 35)	31 dB
Hearos Xtreme Protection (NRR 33)	32 dB
3M 1000/1100 (SNR 37)	33 dB
**Typical ear plug of this type**	**31 dB**

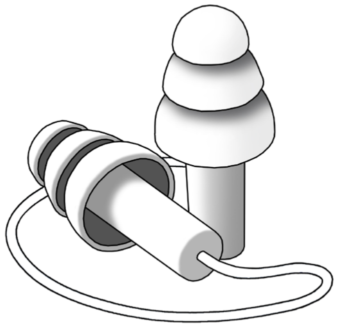

Flanged ear plugs

	Estimated attenuation
3M E-A-R Tri-Flange (SNR 29)	25 dB
Howard Leight Airsoft (SNR 30)	26 dB
Howard Leight Smartfit (SNR 30)	26 dB
3M E-A-R Ultrafit (SNR 32)	28 dB
3M E-A-R Tracer (SNR 32)	28 dB
**Typical ear plug of this type**	**26 dB**

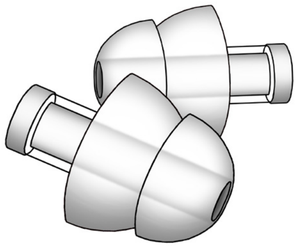

High-fidelity ear plugs

	Estimated attenuation
Etymotic ETY-Plugs (NRR 12)	11 dB
Alpine MusicSafe, gold filter (SNR 18)	14 dB
EarPeace, red filter (SNR 20)	16 dB
Alpine PartyPlug Pro (SNR 21)	17 dB
Eargasm (SNR 21)	17 dB
**Typical ear plug of this type**	**16 dB**

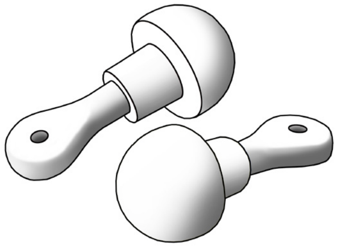

Push-in ear plugs

	Estimated attenuation
3M E-A-R Express Pod Plugs (SNR 28)	24 dB
3M E-A-R Skull Screw (SNR 32)	28 dB
3M No-Touch foam (SNR 35)	31 dB
Howard Leight TrustFit Pod (SNR 36)	32 dB
3M E-A-R Push-Ins (SNR 38)	34 dB
**Typical ear plug of this type**	**31 dB**

High-attenuation earmuffs

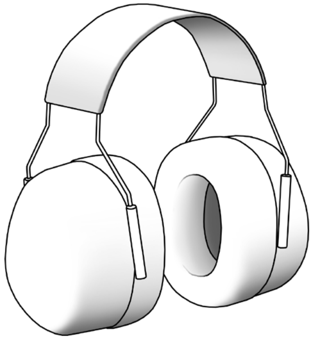

	Estimated attenuation
Mpow (SNR 34)	30 dB
3M Peltor Optime III/105 (SNR 35)	31 dB
3M Peltor X-Series X5 (SNR 37)	33 dB
Fnova 34 dB (NRR 34)	33 dB
Pro For Sho (NRR 34)	33 dB
**Typical earmuffs of this type**	**33 dB**

Banded ear plugs

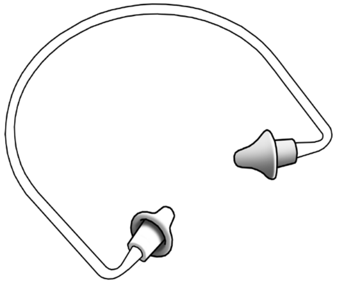

	Estimated attenuation
Moldex 6700 Jazz-Band 2 (SNR 23)	19 dB
Howard Leight QB2HYG (SNR 24)	20 dB
Howard Leight QB1HYG (SNR 26)	22 dB
3M E-A-R Reflex, foam tips (SNR 26)	22 dB
Radians RB2120 RadBand 2 (NRR 25)	24 dB
**Typical ear plugs of this type**	**22 dB**

Low-attenuation earmuffs

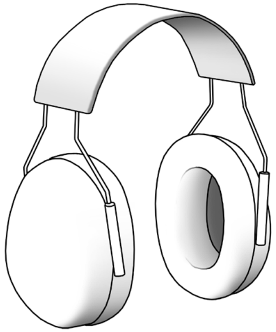

	Estimated attenuation
Howard Leight Impact Sport (NRR 22)	21 dB
3M Peltor Optime I (SNR 26)	22 dB
Silverline 633815 (SNR 27)	23 dB
Neiko 53925A (NRR 26)	25 dB
Silverline 633816 (SNR 30)	26 dB
**Typical earmuffs of this type**	**23 dB**

Other protectors with known SNR or NRR

Hearing protectors with known SNR	Estimated attenuation = **SNR - 4**
Hearing protectors with known NRR	Estimated attenuation = **NRR - 1**
