# Supplementary material for: The Noise Exposure Structured Interview (NESI): An Instrument for the Comprehensive Estimation of Lifetime Noise Exposure
Source: Trends Hear. 2018 Oct 8;22:2331216518803213. doi: 10.1177/2331216518803213 (PMC6176535; doi:10.1177/2331216518803213)
Supplement: Supplemental material4 - Supplemental material for The Noise Exposure Structured Interview (NESI): An Instrument for the Comprehensive Estimation of Lifetime Noise Exposure [file Supplemental_material4.pdf]

NESI Supplementary Material:
Adoption of the Speech Communication Table (SM4)

Source Version of the Speech Communication Table

The basis for the NESI Speech Communication Table is the version reported by Lutman et al. (2008), which has been lightly modified to suit the needs of the NESI (modifications are described below). Ferguson et al. (2018) have since reported evaluation data for a version closely resembling that of Lutman and colleagues, though it specifies six levels of vocal effort, rather than seven, and differs at the highest level of vocal effort. (For Lutman and colleagues, "shouting close to the listener's ear" corresponds to ">110 dBA", whereas for Ferguson and colleagues, ">110 dBA" corresponds to the level at which communication "close to the listener's ear" is "impossible".)

Modifications to the Speech Communication Table

The layout and wording have been altered, so that the expression of communication distance is more straightforward (expressed in words, rather than the use of multiple columns). Dotted lines emphasise the divisions.

Vocal effort is now expressed using verb-adverb phrases (e.g. "talk loudly"), rather than adjective-noun phrases (e.g. "loud voice"), which allows easier expression of the vocal-effort options by the interviewer.

The description of the communication scenario has been altered so that only the listener is free of hearing impairment, not the talker. (The talker in the scenario is the NESI respondent, who might be hearing-impaired, whereas the listener is a *hypothetical* listener with normal hearing.)

The very highest level of vocal effort (shouting into the listener's ear) is now ascribed a sound level of 110 dBA, not >110 dBA as in Lutman et al. (2008). This slightly more conservative estimate more closely resembles that of Ferguson et al. (2018), but was chosen largely for pragmatic reasons. Since exposures at sound levels >110 dBA are rare (Berger et al., 2015), we consider some imprecision in these estimates acceptable.

Evaluation of the Speech Communication Table

Ferguson and colleagues conducted a large-scale evaluation of their version of the Speech Communication Table, using personal dosimetry measurements from 168 employees in 15 workplaces. A summary of results is given in the main NESI paper (see section on Evaluation).

References

Berger, E.H., Neitzel, R., & Kladden, C. (2015). Noise Navigator™ sound level database with over 1700 measurement values (version 1.8). Available at: https://multimedia.3m.com/mws/media/888553O/noise-navigator-sound-level-hearing-protection-database.pdf (accessed 30 April 2018)

Ferguson, M. A., Tomlinson, K. B., Davis, A. C., & Lutman, M. E. (2018). A simple method to estimate noise levels in the workplace based on self-reported speech communication effort in noise. *Submitted for publication*.

Lutman, M.E., Davis, A.C., & Ferguson, M.A. (2008). *Epidemiological evidence for the effectiveness of the noise at work regulations*. Research report, No. RR669. Sudbury, UK: Health and Safety Executive.
