# Supplementary material for: The Noise Exposure Structured Interview (NESI): An Instrument for the Comprehensive Estimation of Lifetime Noise Exposure
Source: Trends Hear. 2018 Oct 8;22:2331216518803213. doi: 10.1177/2331216518803213 (PMC6176535; doi:10.1177/2331216518803213)
Supplement: Supplemental material5 - Supplemental material for The Noise Exposure Structured Interview (NESI): An Instrument for the Comprehensive Estimation of Lifetime Noise Exposure [file Supplemental_material5.pdf]

NESI Supplementary Material:
Estimating Attenuation of Hearing Protection Devices (SM5)

Various types of hearing protection devices (HPDs) are available, differing widely in the attenuation they offer. Those with low-to-moderate attenuation do not guarantee residual sound levels <80 dBA in the ear canal when worn in high-noise environments. Hence, the type of HPD worn by the respondent should be taken into account when quantifying noise exposure.

Since attenuation ratings reported by HPD manufacturers are publicly available, we have sought to derive attenuation estimates from these values. Consequently, attenuation should be easily estimated for protectors reported by future NESI respondents. The table below outlines the basic characteristics of two widely used attenuation ratings and the means by which they are converted into NESI “estimated attenuation”.

	**Single Number Rating (SNR)**	**Noise Reduction Rating (NRR)**
Region of use	The European Union	The United States and elsewhere
Defining standard	ISO 4869-2:1994	ANSI S3.19-1974
Basis for determining the attenuation rating	Mean and standard deviation of real-ear attenuation at threshold, measured for devices fitted by non-expert users	Mean and standard deviation of real-ear attenuation at threshold, measured for devices fitted by expert users
Simplified explanation of the rating calculation	Mean attenuation minus 0.84 standard deviations (i.e. the attenuation achieved by ~80% of users)	Mean attenuation minus two standard deviations (i.e. the attenuation achieved by ~98% of users) minus a 3 dB safety factor
Conversion to NESI “estimated attenuation”	Estimated attenuation<br>= SNR – 4 dB	Estimated attenuation<br>= NRR + 3 dB – 4 dB<br>= NRR – 1 dB

Both conversions above involve subtraction of a 4 dB “derating” value, to account for real-world factors that reduce that reduce the attenuation of HPDs, as recommended by the UK Health and Safety Executive (Brueck, 2009).

The conversion from NRR also involves re-addition of the 3 dB “safety factor” already incorporated in the NRR, to bring the estimate closer in line with that obtained from the SNR. This approach may still be expected to yield a more conservative estimate of attenuation than the SNR, since it reflects the attenuation likely to be achieved by 98% of users, c.f. 80% for the SNR. However, the NRR measurement employs expert rather than naïve users, potentially ameliorating this disparity. In practice, we find that the estimates derived from SNR and NRR seldom differ by more than a few dB. Each is likely sufficiently accurate for the purposes of the NESI, since noise exposure incurred with HPDs contributed relatively little to the overall sound energy of pilot NESI respondents.

References

Brueck, L. (2009). *Real world use and performance of hearing protection*. Research Report RR720. Sudbury, UK: Health and Safety Executive.
