# Supplementary material for: The Noise Exposure Structured Interview (NESI): An Instrument for the Comprehensive Estimation of Lifetime Noise Exposure
Source: Trends Hear. 2018 Oct 8;22:2331216518803213. doi: 10.1177/2331216518803213 (PMC6176535; doi:10.1177/2331216518803213)
Supplement: Supplemental material6 - Supplemental material for The Noise Exposure Structured Interview (NESI): An Instrument for the Comprehensive Estimation of Lifetime Noise Exposure [file Supplemental_material6.pdf]

NESI Supplementary Material: Quantifying Firearm Noise Exposure (SM6)

To account for the greater auditory hazard posed by impulsive sound, NESI has adopted the kurtosis-corrected noise metric of Goley et al. (2011):

$L'_{Aeq} = L_{Aeq} + 4.02 * \log_{10} (\beta / \beta_G)$

where L'_{Aeq} is kurtosis-corrected A-weighted equivalent continuous level, L_{Aeq} is uncorrected A-weighted equivalent continuous level, 4.02 is a constant derived from dose-response data in chinchillas, β is the kurtosis statistic of the noise, and β_G is the kurtosis statistic for Gaussian noise ($\beta_G = 3$).

For impulses whose acoustic energy expressed as A-weighted equivalent continuous *8-hour* level (L_{Aeq8hr}), the equation becomes:

$L'_{Aeq8hr} = L_{Aeq8hr} + 4.02 * \log_{10} (\beta / \beta_G)$

Incorporation of firearm noise into the NESI can therefore be achieved by combining L_{Aeq8hr} and β for firearm impulses measured at the shooter's ear. Flamme et al. (2009) and Meinke et al. (2014) have reported these data for a wide variety of firearms. Due to a markedly bimodal distribution of L_{Aeq8hr}, we have elected to dichotomize these weapons into low-calibre (.22 and .17) rifles and all other hand-held firearms (excepting air guns). A single kurtosis correction factor has been applied to both categories, since this factor was found to differ little between firearms:

	**Low-calibre (.22 and .17) rifles**	**Other hand-held firearms (except air guns)**
Energy of a round, expressed as L_{Aeq8hr}	Mean = **66.7 dBA**, SD = 2.5,<br>n = 5 (Meinke et al., 2014)	Mean = **81.8 dBA**, SD = 3.5,<br>n = 21 (Meinke et al., 2014)
Kurtosis statistic	Median = 78.5, range = 20 to 220, n = 10 (Flamme et al., 2009)	
Kurtosis correction factor	Kurtosis correction factor = $4.02 * \log_{10} (\beta / \beta_G)$<br>For the firearms reported by Flamme et al. (2009), mean kurtosis correction factor = **5.4 dBA** (SD = 1.2)	
Kurtosis-corrected A-weighted equivalent continuous 8-hour level (L'_{Aeq8hr})	L'_{Aeq8hr}<br>$= L_{Aeq8hr} + 4.02 * \log_{10} (\beta / \beta_G)$<br>= 66.7 + 5.4<br>= **72.1 dBA**	L'_{Aeq8hr}<br>$= L_{Aeq8hr} + 4.02 * \log_{10} (\beta / \beta_G)$<br>= 81.8 + 5.4<br>= **87.2 dBA**
Conversion to NESI units of noise exposure	NESI units of noise exposure<br>$= 10^{(\text{level} - 90)/10}$ x hours / 2080<br>$= 10^{(72.1 - 90)/10}$ x 8 / 2080<br>= 0.000062 ≈ **1 / 16000**	NESI units of noise exposure<br>$= 10^{(\text{level} - 90)/10}$ x hours / 2080<br>$= 10^{(87.2 - 90)/10}$ x 8 / 2080<br>= 0.002213 ≈ **1 / 500**

Note that correcting for kurtosis in the above calculations involves adding 5.4 dB to the equivalent continuous level. Interestingly, Murphy and Kardous (2012) remark that "for occupational noise exposures with impulsive content, the rule of thumb is to add 5 dB to the continuous dose estimate to compensate for the increased risk" – an adjustment similar to that obtained via Goley and Kim's kurtosis statistic.

Exceptions

We recommend that exposures incurred while wearing hearing protection be disregarded, due to their very low sound energy. Assuming protector attenuation of 30 dB, exposure to ~500000 such rounds would be required to accrue a single NESI noise unit.

Exposures to air rifles should also be disregarded, since all rifles tested by Lankford et al. (2016) were associated with a shooter-ear L_{Aeq8hr} below 54 dBA, yielding less than a billionth of a NESI noise unit.

Exposure to impulse noise from sources other than hand-held firearms (e.g. artillery and blast noise) is beyond the scope of the NESI.

Potential Limitations and Alternative Methods

It is important to note that our energy-based metric represents a single approach to the quantification of firearm noise exposure, which may not agree with other measures of auditory hazard. For example, in UK law, the threshold beyond which employers must provide hearing protection is a peak level of 135 dBC ***or*** a daily average level of 80 dBA (Health and Safety Executive, 2005). A low-powered Remington 514 rifle produces a peak sound level of 139.6 dBA, but its energy, expressed as L_{Aeq8hr}, is only 63.8 dBA (Meinke et al., 2014). Even after adding a 5.4 dB correction for kurtosis, as in the NESI, 12 rounds could be fired from the Remington before the corrected L_{Aeq8hr} exceeded 80 dBA. Hence, it is plausible that our energy-based approach might under-weight firearm noise. At present, all that can be confidently stated is that energy-based metrics of impulsive noise are increasingly advocated as a basis for firearm-noise damage risk criteria (e.g. Murphy and Kardous, 2012), and that correcting energy-based metrics of impulsive noise for kurtosis improves their ability to predict noise-induced auditory damage (e.g. Zhao et al., 2010). In short, kurtosis-corrected energy appears a plausible metric, but would surely benefit from further evaluation.

Future NESI users may wish to distinguish between different types of hand-held firearm when quantifying exposure, rather than accepting the dichotomous categorisation used here. Such users should obtain the equivalent continuous level of a round fired from the weapon in question, as measured at the shooter's ear. They may then combine it with the 5.4 dB kurtosis correction factor and convert into NESI units of noise exposure, as demonstrated in the above calculations.

Another potential limitation is our exclusion of exposures incurred while wearing hearing protection. If HPDs worn during firearm exposure provide substantially less than 30 dB of attenuation, then such exposures might make a meaningful contribution to lifetime noise exposure. If users wish to incorporate these exposures into the NESI, they may obtain an estimate of the attenuation provided by the equipment and use it to modify the equivalent continuous level, before combining this value with the kurtosis correction factor and converting to NESI units of noise exposure.

References

Flamme, G. A., Wong, A., Liebe, K., & Lynd, J. (2009). Estimates of auditory risk from outdoor impulse noise. II: Civilian firearms. *Noise & Health*, *11*(45), 231–242. https://doi.org/10.4103/1463-1741.56217

Goley, G. S., Song, W. J., & Kim, J. H. (2011). Kurtosis corrected sound pressure level as a noise metric for risk assessment of occupational noises. *The Journal of the Acoustical Society of America*, *129*(3), 1475–1481. https://doi.org/10.1121/1.3533691

Health and Safety Executive (2005). *Controlling noise at work: The Control of Noise at Work Regulations 2005*. Norwich: HSE Books.

Lankford, J.E., Meinke, D.K., Flamme, G.A., et al. (2016). Auditory risk of air rifles. *International Journal of Audiology* 55 Suppl 1: S51-58. DOI: [10.3109/14992027.2015.1131851](https://doi.org/10.3109/14992027.2015.1131851).

Meinke, D.K., Murphy, W.J., Finan, D.S., et al. (2014). Auditory risk estimates for youth target shooting. *International Journal of Audiology* 53 Suppl 2: S16-25. DOI: [10.3109/14992027.2013.865845](https://doi.org/10.3109/14992027.2013.865845).

Murphy, W.J., & Kardous, C.A. (2012). *A case for using A-weighted equivalent energy as a damage risk criterion*. In-depth survey report for the Engineering and Physical Hazards Branch, No. 350–11a, 10 January 2012. Cincinnati: National Institute for Occupational Safety and Health.
